# Intraoperative Recurrent Laryngeal Nerve Monitoring During Pediatric Cardiac and Thoracic Surgery: A Mini Review

**DOI:** 10.3389/fped.2020.587177

**Published:** 2020-11-27

**Authors:** Claire M. Lawlor, Benjamin Zendejas, Christopher Baird, Carlos Munoz-San Julian, Russell W. Jennings, Sukgi S. Choi

**Affiliations:** ^1^Department of Otolaryngology, Children's National Health System, Washington, DC, United States; ^2^Department of Surgery, Boston Children's Hospital, Boston, MA, United States; ^3^Department of Cardiac Surgery, Boston Children's Hospital, Boston, MA, United States; ^4^Department of Anesthesiology, Critical Care, and Pain Medicine, Boston Children's Hospital, Boston, MA, United States; ^5^Department of Otolaryngology and Communication Enhancement, Boston Children's Hospital, Boston, MA, United States

**Keywords:** pediatric cardiac surgery, recurrent laryngeal nerve (RLN), recurrent laryngeal nerve injury, recurrent laryngeal nerve monitoring, tracheoesophageal fistula (TEF), esophageal atresia (EA), tracheobronchomalacia (TBM)

## Abstract

**Objective:** Review techniques for intraoperative recurrent laryngeal nerve (RLN) monitoring during pediatric surgery for esophageal atresia, tracheoesophageal fistula, tracheobronchomalacia, and cardiac surgery.

**Summary Background Data:** Literature was reviewed for reports of intraoperative recurrent laryngeal nerve monitoring in cervical, thoracic, and cardiac surgical procedures which place the RLNs at risk for injury.

**Methods:** Review paper.

**Results:** The RLN is at risk during pediatric surgery for esophageal atresia, tracheoesophageal fistula, tracheobronchomalacia, and cardiac surgery. Intraoperative nerve monitoring has decreased rates of RLN injury in thyroid surgery. Intraoperative RLN monitoring techniques appropriate for pediatric surgery are discussed, including endotracheal tubes with integrated surface electrodes, adhesive surface electrodes for smaller endotracheal tubes, endolaryngeal electrodes, and automatic periodic continuous intra-operative stimulation.

**Conclusions:** Multiple techniques exist to monitor the RLN in children undergoing cervical, cardiac, and thoracic surgery. Monitoring the RLN during procedures that place the RLNs at risk may help decrease the rate of RLN injury.

## Brief Historical Perspective

The recurrent laryngeal nerves (RLNs) are branches of the vagus nerves that carry motor, sensory, and parasympathetic fibers to the larynx. The right and left vagus nerves run within the carotid sheath from the skull base into the mediastinum. On the left (in typical anatomy), the RLN diverges from the vagus and loops medially around the aorta posterior to the ductus arteriosus to ascend toward the larynx variably but close to the tracheoesophageal groove. On the right (in typical anatomy), the RLN diverges from the vagus nerve at the level of the right subclavian artery and its course toward the larynx is more lateral to the trachea-esophageal groove. The right RLN loops around the subclavian artery, ascends along the apical pleura until it reaches the common carotid artery, where it runs posteriorly until it enters the tracheoesophageal groove near the cricothyroid joint and innervates the larynx. In <1% of patients, the right RLN is non-recurrent and diverges directly from the vagus at the level of the thyroid gland (1, 2). These non-recurrent nerves are almost always associated with other anatomic anomalies such as an aberrant (retroesophageal and/or retrotracheal) subclavian artery. Non-recurrent laryngeal nerves are at risk of iatrogenic injury when not recognized intraoperatively (3). Occasionally the right RLN wraps around the aberrant subclavian artery in the chest, indicating that there is not perfect correlation between RLN pathway and vascular anomalies. Reliably, the right RLN wraps around the right aortic arch in patients with right aortic arch and double aortic arch, while the left RLN reliably wraps around left sided ductal ligament even in cases of right aortic arch. These anatomic variations must be understood as they place both RLNs at risk of injury during surgery in the neck and mediastinum. Even when the RLNs follow their conventional course there are fine variations in anatomy between patients, including relationships to thyroid vasculature, parathyroid glands, and distal branching. However, the vagus nerve giving off the left recurrent laryngeal nerve which then passes around the ligamentum arteriosum is a very useful operative landmark.

Injury to the RLN can occur from mechanisms such as stretch, heat, electrocautery, compression, inadvertent transection, and compromise of vascular supply (1). Following RLN injury, ensuant vocal fold immobility (VFI) and loss of laryngeal sensation results in significant morbidity for pediatric patients, including respiratory distress (typically inspiratory stridor), feeding difficulty due to aspiration and loss of sensation, recurrent respiratory infections, and voice changes (4). The clinical course for patients with iatrogenic RLN injury can vary greatly and depends on whether the injury results in unilateral or bilateral immobility. The rate of spontaneous recovery after iatrogenic injury is ~25–46% and may occur as long as 4 years after the insult (4, 5). When resolution does not occur, additional interventions may be indicated, ranging from diet modification to injection medialization, thyroplasty, tracheotomy, and gastrostomy tube placement (4). Persistent dysphonia can have both social and academic impact on pediatric patients (6). Iatrogenic bilateral VFI is a grave complication. The primary symptoms are dyspnea, stridor, dysphagia, and dysphonia. Historically, nearly all pediatric bilateral VFI patients required a tracheotomy for airway protection. More recent conservative measures have resulted in a tracheotomy rate of 57–69% of these patients (7).

**Figure 1:**
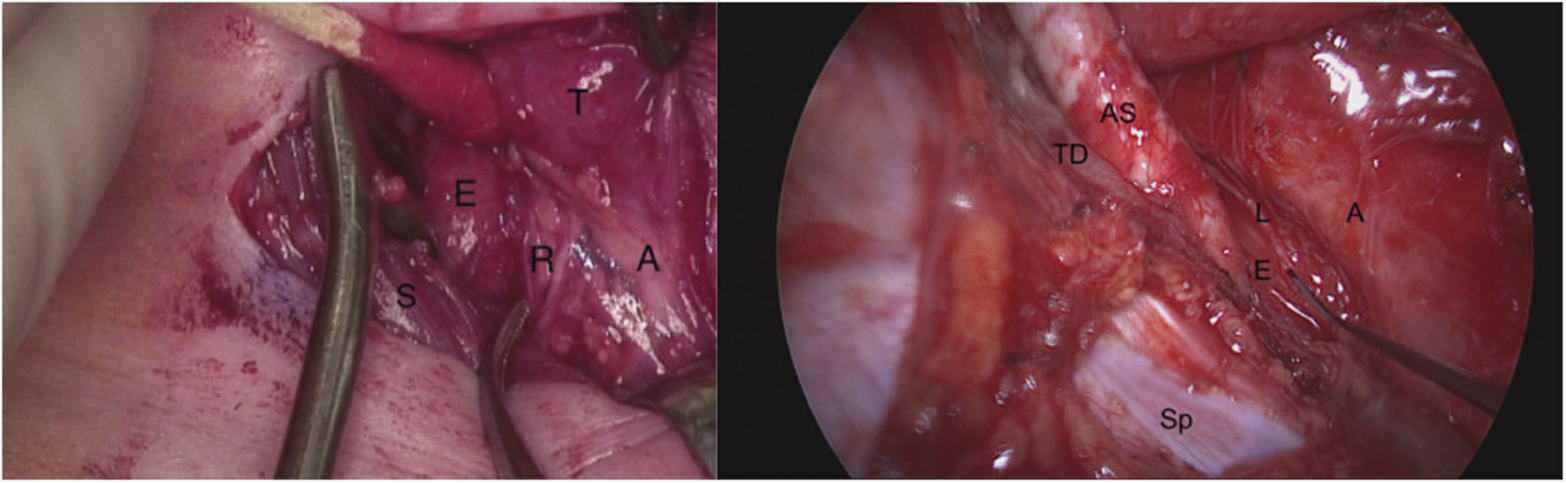
On the left: Right neck dissection during tracheoesophageal fistula repair demonstrating the right recurrent laryngeal nerve in the tracheoesophageal groove branching before it enters the larynx. T, thyroid gland; A, airway (trachea); S, sternocleidomastoid muscle; E, esophagus; R, two branches of right recurrent laryngeal nerve. On the right: The left recurrent laryngeal nerve seen from posterolateral direction during a right thoracotomy (right non-recurrent laryngeal nerve not shown). The probe is pointing to the left recurrent laryngeal nerve as it lies on the thoracic esophagus. AS, aberrant right subclavian artery; TD, thoracic duct; E, esophagus; A, airway (trachea and bronchi); Sp, spine; L, left recurrent laryngeal nerve.

RLN injury during surgery for esophageal atresia (EA), tracheoesophageal fistula (TEF), and tracheobronchomalacia (TBM) is established in the literature as the RLN often courses through the operative field (Figure 1). The incidence ranges from 11 to 50% and varies by approach, the patient's anatomy, and concurrent cardiac procedures (8–13). Identifying and protecting the RLN is particularly difficult in the surgery of neonates and in revision cases (13). Similarly, there are a number of associations of RLN injury with heart disease and cardiac surgery. Compressive injury between the left pulmonary artery and aorta is frequently associated with thoracic aortic aneurysms (14) and left atrial enlargement associated with mitral stenosis can compress the RLN against the arch (15, 16). During cardiac surgery intra-operative hypothermia and local cooling with ice slush have also been reported to cause RLN injury and hoarseness (17, 18). Due to the proximity of the RLN, interventions related to patent ductus arteriosus (PDA) like clip or suture ligation, or coil implantation can potentially damage the RLN (19–21). Prevalence of left vocal cord paralysis after PDA ligation has been reported as 4.2% of the whole population, and 8.0% in low-birth-weight infants (19). The incidence may be under-reported due to failure to diagnosis transient weakness or hypomobility and lack of consistent pre- and postoperative evaluation of the vocal cord mobility. This represents an area of future investigation. Injury to the recurrent laryngeal nerves contributes to poor long-term swallowing outcomes and may trade one vexing airway lesion (TBM) for another (VFI) (9, 12, 13).

Intraoperative nerve monitoring (IONM) provides immediate feedback to the surgeon on RLN location and function during surgery. IONM yields three surgical advantages: identification of the RLN, aid in dissection, and prognostication of post-operative neural function and lesion site identification (22). In adult patients, IONM demonstrates a 99% negative predictive value and 75% positive predictive value of intraoperative nerve signaling loss and post-operative vocal fold paralysis (23).

IONM of the RLNs is standard of practice in adult thyroidectomy (22). It is also employed in some thoracic cases such as surgery of the esophagus, lung, and mediastinum (24–26). IONM has not yet been widely adopted in pediatric surgery. The use of nerve monitoring in children was first described in 2002 and its use in pediatric surgery is expanding (27). IONM has been described in pediatric thyroidectomy (28–30) and tracheal surgery (31). A case report of IONM use during H type TEF has been published (32). Trials for use of IONM in pediatric cardiac and thoracic surgery are ongoing. Routine utilization of IONM in pediatric surgery has faced challenges including adaptation of the equipment to pediatric patients, the need to alter the anesthetic management of the patient during surgery and training of the surgeons on the use of IONM. We review the current techniques available for pediatric IONM in surgery for EA, TEF, and TBM which hopefully will enable surgeons to use IONM consistently during surgery.

## Available Techniques

There are several current options for IONM during cervical and thoracic surgery, including surface electrodes on endotracheal tubes, endolaryngeal electrodes, and APS (automatic periodic stimulation) monitoring. For any of the techniques discussed, imperative to successful IONM is preoperative discussion with the anesthesia team and avoidance of chemical paralysis. The anesthesia technique chosen, in addition to avoiding muscle relaxant, must allow the safe conduct and good exposure of the surgical field for the success of the procedure. The earliest IONM techniques described in pediatric patients was for thyroid surgery. Transligamentous electrodes were placed though the cricothyroid membrane into the vocalis muscle (27, 33). While this approach remains available, it is often not practical during surgery for EA, TEF, TBM, or cardiac surgery because the leads would be in the operative field. Table 1 summarize the pros and cons of the IONM techniques described in this review.

**Table 1:** Pros and cons of IONM techniques.

**IONM Technique**	**Pros**	**Cons**
Endotracheal tube with integrated surface electrodes	No slippage of surface electrodes Commercially available	Smallest size is 5.0 mm ID Improper placement of electrodes Shifting of electrodes with positioning of patient May only alert surgeon after injury to nerve has occurred Noisy signal feedback Intermittent stimulation only
Endotracheal tube with adhesive surface electrodes	Can apply single-channel electrodes to ETT as small as 2.0 mm ID and monitor much smaller, younger patients Confirms nerve stimulation at close of case	Same as above for integrated surface electrodes Surgeon or anesthesia must apply electrodes to ETT Adhesive electrodes may slip during surgery
Endolaryngeal hookwire electrode	May be used in patients of any age Enhanced monitoring sensitivity over surface electrodes on ETT Smaller, more delicate electrode than paired electrode Confirms nerve stimulation at close of case	Electrodes must be inserted into the vocalis muscle under direct laryngoscopy May be displaced intraoperatively Difficult airway exposure may limit placement Risk of airway foreign body Risk of laryngeal trauma, edema, and hematoma May only alert surgeon after injury to nerve has occurred Noisy signal feedback Intermittent stimulation only
Endolaryngeal prass paired electrode	Larger electrode than hookwire, more difficult to displace intraoperatively Confirms nerve stimulation at close of case	Same as above for endolaryngeal hookwire electrode Larger electrode, risk of greater laryngeal trauma
Automatic Periodic Stimulation Electrode	Provides continual feedback on nerve function Provides real-time feedback on nerve injury Confirms same pre- and postoperative latency and amplitudes of nerve function	Must dissect in the carotid sheath for placement Size of electrode may preclude use in very young children Possible undesirable vagal stimulation Same as above for ETT surface electrodes

*IONM, intraoperative nerve monitoring; ETT, endotracheal tube*.

### Endotracheal Tubes With Integrated Surface Electrodes

Endotracheal tubes with integrated electromyography (EMG) electrodes are available commercially for IONM, including the Medtonic NIM TriVantage EMG Tube (Medtronic, Jacksonville, Florida) and the Neurovision Cobra EMG Endotracheal Tube (Neurovision Medical Products, Ventura, California). We will discuss the Medtronic system as an example as both products are similar. The NIM nerve monitoring system continuously monitors EMG activity from each RLN individually. The electrodes are wired into the surface of the endotracheal tube; no EMG needle placement into the patient is required. The patient is intubated with the NIM TriVantage endotracheal tube and connected to NIM-Neuro 3.0 Monitoring System. With proper placement, the EMG electrodes are in close proximity to the true vocal folds bilaterally and continually track the EMG activity of the laryngeal muscles. Thus, when RLN is stimulated in the surgical field, the NIM system provides the surgeon with immediate audible and visual feedback as well as quantifiable EMG data (34). The RLN does not need to be identified for stimulation to occur; dissection near the nerve may cause stimulation. Alternatively, a nerve stimulator may be used by the surgeon to intentionally stimulate the nerve and confirm its identity. IONM in this manner is common practice in adult thyroid surgery (22). Use of endotracheal tubes with integrated surface electrodes has also been described in pediatric thyroid surgery (30, 35). At the conclusion of the procedure, the RLN can be stimulated to confirm the continuity of the nerve.

Limitations of the Medtronic NIM TriVantage EMG tubes includes their size: currently, the smallest endotracheal tube available is cuffed with a 5.0 mm inner diameter, 6.5 mm outer diameter, appropriate for a child aged 4 years and older. Challenges inherent to IONM in this fashion include improper placement of the EMG electrodes (e.g., too proximal, too distal, or rotated) and poor detection of vocal fold stimulation, providing the surgeon with a false negative response and no ability to monitor RLN function which can result in unexpected post-operative nerve injury. The endotracheal tube may also move with manipulation of the patient's head and neck even after adequate placement is confirmed during laryngoscopy. Additionally, if the nerve is accidentally transected, the surgeon may only notice after the injury when the audible and visual warning is elicited from the NIM system. Conversely, the system may register stimulation of the nerve when the surgeon is not operating near the nerve (false positive) or provide noisy signal feedback inhibiting accurate monitoring.

### Endotracheal Tubes With Adhesive Surface Electrodes

Nerve monitoring electrodes can also be fixed to generic endotracheal tubes by the surgeon, otolaryngologist, or anesthesiologist, such as the Neurovision Dragonfly Stick on Electrode (Neurovision Medical Products, Ventura, California). These systems function similarly to the systems for the integrated surface electrode tubes and are designed to work with Neurovision's Nerveana Nerve Locator and Monitor system but are also compatible with the Medtronics NIM 3.0 system. An appropriate endotracheal tube is selected for the patient based on their age and size. The tube is straightened, and the adhesive electrode is wrapped around the distal endotracheal tube, and the patient is intubated with the electrode positioned at the vocal folds. Thereafter, the adhesive surface electrodes function similarly to the integrated surface electrodes with the same inherent benefits and challenges. In addition to the risk electrode displacement due to head positioning after intubation, the adhesive electrodes may slip after exposure to airway secretions. This may result in false negatives on the nerve monitoring system. The Dragonfly electrodes are available in single channel electrode and double channel electrode kits. The single channel kits are available for endotracheal tubes (ETT) size 2.0–10.0, making them very versatile for use in patients of all ages. They require downsizing the ETT to avoid excess laryngeal pressure. The double channel electrodes are available for endotracheal tube sizes 6.0–9.5, thus only available for older children and young adults. However, it is possible to trim the double channel electrode and use it on an ETT as small as size 3.0. Single channel electrodes monitor both vocal folds simultaneously and do not provide the surgeon with the laterality of nerve stimulation. Double channel electrodes will distinguish stimulation of each RLN individually. The Dragonfly adhesive electrodes may be applied to cuffed and uncuffed endotracheal tubes (36). Adhesive surface electrodes on endotracheal tubes have been used successfully in children as young as 2 months of age (30).

### Endolaryngeal Hookwire Electrode

Endolaryngeal EMG hookwire electrodes, such as those available from Medtronic, have been used to successfully monitor the RLN in pediatric thyroid surgery (37). After the patient is intubated, a direct laryngoscopy is performed. Under direct, microscopic, or telescopic visualization, the hookwire electrodes are placed into the vocalis muscles of the glottis bilaterally. The leads are then secured externally to the patient's skin and connected to the monitoring system, such as the NIM, which functions as previously described. Intraoperatively, when the nerve is stimulated intentionally via the nerve probe or during the dissection, the movement (EMG) of the vocalis will be detected by the electrodes and the NIM system alerts the surgeon. The advantages of this system include the ability to apply the electrodes and perform IONM in patients of any age. Studies have suggested that endolaryngeal EMG hookwire electrodes enhance sensitivity for RLN stimulation over surface electrodes on an endotracheal tube (38, 39). Disadvantages include the time and technical skill required to set up the EMG hook wires. Depending on level of comfort, the electrodes may be placed by the surgeon or in partnership with otolaryngology colleagues. If the patient has a difficult airway to expose on direct laryngoscopy, this limits the ability to place the electrodes. The electrodes can be removed accidentally during the procedure. Care must be taken to examine the hookwire electrodes after removal to ensure that they are intact and no foreign body was left in the airway. As these electrodes are wires placed into the patient's muscle, there is theoretical risk of laryngeal trauma, edema, hematoma, and bleeding.

### Endolaryngeal Prass Paired Electrode

A Prass paired electrode can be used in a similar fashion to the hookwire electrode. Medtronic manufactures Prass paired electrodes that are compatible with the NIM system. Direct laryngoscopy is used to expose the larynx and the double-pronged electrode is placed just lateral to the vocal cords into the vocalis. The leads are secured to the patient externally. Advantages of the Prass paired electrode over the hookwire electrode include its sturdiness. We have found the larger electrode is less likely than the hookwire electrodes to pull out of position intraoperatively but is technically difficult to place due to its bulkiness. The Prass paired electrode is also less likely to fracture and result in a foreign body. The disadvantages are similar to the hookwire electrode.

### Automatic Periodic Stimulation Electrode

The Automatic Periodic Stimulation (APS) Electrode (Medtronic) provides continuous real-time stimulation and monitoring of the vagus nerve, and thus the RLN. The patient is intubated with an endotracheal tube with surface electrodes and these are connected to the NIM monitoring system. A cervical approach is used to access the carotid sheath. The vagus nerve is identified and confirmed with hand-held nerve stimulator. A 1–2 cm segment of nerve is dissected free from surrounding structures. The APS electrode is carefully placed around the vagus nerve proximal to the RLN take off. The electrode is then connected to the NIM monitoring system. The electrode in the neck delivers periodic 1–2 mA stimulation every 2 s to the vagus nerve that is detected by the NIM surface electrodes on the endotracheal tube (40). Baseline EMG latency and amplitudes are automatically calibrated. APS detects small changes in these variables and provides early warning when possible injury occurs. As in conventional IONM, the surgeon may use a nerve probe to intermittently stimulate the nerve to confirm its location or function. Electrode responses may be monitored by the NIM system or by an electrophysiologist present in the OR. The APS system provides feedback on the integrity of the RLN regardless of identification of the RLN intraoperatively.

APS differs from conventional IONM techniques in that APS provides continual feedback on nerve function whereas there is only intermittent stimulation by a stimulating probe when using other methods (41). APS can alert the surgeon in real time when EMG latency and amplitude are affected by intraoperative events, such as traction on or dissection on the RLN, and allows for immediate corrective action. At the close of the case, the APS system can be used to confirm that RLN latency and amplitudes have not changed, indicating that no RLN injury occurred intraoperatively. An additional benefit of the APS system is the ability to determine reassuring nerve function after an initial, unilateral dissection. If the APS system determines that the latency and amplitude of the RLN is decreased following a unilateral dissection, the surgeon may stage dissection on the contralateral side or be even more vigilant to prevent the more significant complication of bilateral RLN compromise.

APS IONM has been described extensively in adult thyroid surgery (42, 43). Use of the APS system has not yet been reported in children. The advantage over conventional, intermittent IONM as described in the previously mentioned techniques is the ability to provide dependable feedback quickly enough that the surgeon is able to act before the RLN is damaged intraoperatively (42, 43). This is superior to conventional IONM, which alerts the surgeon only after the nerve has been injured. The APS system has the potential to elicit undesirable vagal stimulation, causing autonomic nervous system imbalance such as cardiac (arrhythmias, bradycardia), pulmonary (bronchospasm), gastrointestinal (nausea, vomiting), and central (headaches) effects. The APS system also requires laryngeal electrodes, typically surface electrodes on an endotracheal tube, thus the risks/limitations of those methods in children also apply. The greatest limitation is electrode placement and the risks inherent to dissecting within the carotid sheath. Bilateral nerve monitoring requires bilateral neck dissections. If a cervical approach is not planned for the procedure, this IONM technique is of low utility or would necessitate a separate neck dissection. The APS system requires that the APS electrode and the vagus nerve be similar in size to facilitate adequate stimulation; this may preclude the use of this system in very young children.

## Future Directions

A single report of IONM during a pediatric TEF repair has been published (32). Unlike adult thyroid surgery, there is not a systematic approach to avoiding RLN injury during cardiac and thoracic surgery in infants and children. It is vital to increase awareness of the morbidity associated with RLN injury amongst pediatric surgeons. Consistent documentation of pre- and postoperative vocal fold function via flexible fiberoptic laryngoscopy in collaboration with otolaryngology colleagues will delineate the incidence of RLN injury in surgery for EA, TEF, TBM, and cardiac surgery. Furthermore, greater implementation of IONM in pediatric surgery may decrease iatrogenic RLN injury in these cases.

Optimization of airway and swallowing outcomes following surgery for EA, TEF, TBM, and cardiac surgery is already an area of active investigation. A greater focus on the role of RLN injury in the postoperative course will improve patient results. Innovation is needed to develop better means of monitoring the RLN in very small infants. Further investigation into the risks and benefits of the IONM techniques described here, including optimal means of monitoring based on the patients age, weight, or specific procedure, would be of great benefit to surgeons performing these cases. Finally, advances in continuous IONM, including devices similar to the APS but applicable in thoracic surgery without a neck dissection, will continue to advance the surgeon's ability to monitor and protect the RLN during these complex cases.

## Author Contributions

All authors listed have made a substantial, direct and intellectual contribution to the work, and approved it for publication.

## Conflict of Interest

The authors declare that the research was conducted in the absence of any commercial or financial relationships that could be construed as a potential conflict of interest.
